# Effects of Supplementing Grape Pomace to Broilers Fed Polyunsaturated Fatty Acids Enriched Diets on Meat Quality

**DOI:** 10.3390/ani10060947

**Published:** 2020-05-29

**Authors:** Raluca Paula Turcu, Tatiana Dumitra Panaite, Arabela Elena Untea, Cristina Șoica, Mădălina Iuga, Silvia Mironeasa

**Affiliations:** 1 Chemistry and Nutrition Physiology Department, National Research and Development Institute for Biology and Animal Nutrition, 077015 Balotesti, Romania; tatiana_panaite@yahoo.com (T.D.P.); arabela.untea@ibna.ro (A.E.U.); soicacristina4@gmail.com (C.Ș.); 2 Faculty of Food Engineering, Ştefan cel Mare University, 720229 Suceava, Romania; madalina.iuga@usm.ro (M.I.); silviam@fia.usv.ro (S.M.)

**Keywords:** broiler, grape pomace, meat quality, color, texture, lipid, peroxidation

## Abstract

**Simple Summary:**

Chicken meat and its products are among the first-choice foods in most countries, due to the health benefits they provide and the relatively low price. However, polyunsaturated fatty acids (PUFA) are highly susceptible to peroxidation, affecting nutritional, sensory characteristics and meat shelf life. A reasonable way and a good tactic to delay their lipid peroxidation would be using a natural antioxidant in feed formulation. Grape pomace (GP) is an agro-industrial by-product with high nutritional value as it is a rich source of polyphenols. The supplementation of broiler diets with grape pomace indicated an intensified color, improved texture and a decrease of lipid peroxidation parameters of broilers’ meat that had been fed with PUFA enriched diets.

**Abstract:**

The effects of using grape pomace as natural antioxidant in polyunsaturated fatty acids enriched broiler diets (4% flaxseed meal) on color, texture and lipid peroxidation of meat were evaluated. The 4-week feeding trial was conducted on 200, Cobb 500 broilers, assigned to 5 groups and housed in an experimental hall with floored pens. Compared to the control group, the diet for the experimental groups included 3% or 6% of two grape pomace varieties, white and red. Diet formulation enrichment with red grape pomace influenced the meat color parameters, underlining an accentuated total color difference (ΔE) for both breast and thigh as compared to the control. The dietary supplementation with grape pomace led to the increase of meat hardness regardless of the amount and variety added. The grape pomace inclusion in broiler diets positively influenced meat color and texture. Regarding the lipid oxidation parameters, there was a decrease of the value of thiobarbituric acid-reactive substances (TBARS) in thigh meat in all experimental groups. Additionally, significant differences were highlighted for breast in 3% white grape pomace and 6% red grape pomace supplemented groups when compared to the control group. In conclusion, the supplementation of broiler diets enriched in PUFA with grape pomace improved meat color and texture, which are essential parameters for consumer’s choice. Also, the grape pomace supplementation indicated an improvement of thigh meat oxidative stability, especially regarding the TBARS value.

## 1. Introduction

During the last few decades, food quality and safety have been the subject of many debates in various fields, such as industry or research. Today’s consumers pay more attention to food choices because they are becoming more informed and therefore more exigent when buying a product. Thus, in order to meet the needs of consumers, differentiating the quality of food has become fundamental, leading to an increase in global competition on the food and production market [1].

Chicken meat and its by-products are among the first-choice foods in most countries, due to the health benefits they provide [2] and the relatively low price [3]. According to the National Chicken Council, the price of chicken is about 2 or 3 times lower, than that of beef or pork. Poultry industry has long sought strategies to improve the production parameters, to the detriment of meat final quality [4]. Therefore, recent research has focused on obtaining meat that has beneficial effects on health, to prevent the occurrence of diseases. Polyunsaturated fatty acids (PUFA) n-3 and n-6 are essential fatty acids for both human and animal diet. The problem facing consumers is that of the high intake of n-6 fatty acids diet, compared to the n-3 diet [5]. Enriching the broiler diets, using vegetable oil sources of PUFA n-3 is an effective method of increasing the content of essential fatty acids in meat. A direct relationship was reported between n-3 fatty acid content and the values of thiobarbituric acid-reactive substances (TBARS) determined in liver [6] as PUFA are highly susceptible to peroxidation, a process that affects both the quality of feed and the obtained meat [7]. The nutritional, sensory characteristics and meat shelf life are highly affected by oxidative rancidity which may negatively influence consumers’ acceptability [8].

The strategies employed lately have aimed to include the natural antioxidants in broiler diets in order to improve their oxidative stability and consequently of the meat broiler [9]. Compounds with antioxidant activity have both in vivo and post-mortem beneficial actions against oxidative stress and oxidative rancidity in meat production and food industry, respectively [10]. Antioxidants are effective on broilers’ health and welfare but they also contribute to meat yield and quality parameters improvement mostly for broilers grown in intensive systems [8]. Some studies presented potential beneficial antioxidant activity of plant extracts and essential oils added in broiler diets and its effects on meat quality [11,12].

Grape pomace (GP) is an agro-industrial by-product with high nutritional value as it is a rich source of polyphenols [13]. The most important polyphenols that are present in grape pomace are anthocyanins and resveratrol, which are known as cardio-protective nutrients [14]. As a means of improving the feed quality and implicitly the broiler meat through nutrition, the use of grape pomace in the fed formulation, is a natural way to delay their lipid peroxidation, especially if they have been enriched in polyunsaturated fatty acids (PUFA). The natural antioxidants inclusion in broiler diets delays the oxidative process of meat, thus prolonging its shelf life [15]. In vivo and in vitro studies performed in recent years [16,17,18] have shown the beneficial effects of grape pomace supplementation in broiler diets.

There are many factors that influence meat quality but two of the most important quality attributes are appearance and texture. Appearance, namely the color and the color variations of meat, is an important quality parameter for consumers because it is responsible for the initial selection of the product and for the final satisfaction [19]. Depending on the color, meat can be classified as compliant, with normal appearance and considered appetizing. As well as the color, texture represents a sensory characteristic of meat that influences the perception of the consumer regarding the quality of meat. The evaluation of texture profile allows establishing several parameters relevant to the quality of meat, using objective methods [20]. There are several factors, such as age, sex or defrost time that can influence the final texture of chicken meat. At the same time, the diameter and localization of muscle fibers can influence the texture of meat [20]. Avoiding defects in appearance that could negatively influence consumer perception or product price has become essential [21,22], color and texture thus being two determinants for the economic value of meat [23].

The objective of this study was to improve the quality attributes for consumers and oxidative stability of meat by using grape pomace as a natural antioxidant in broiler diets enriched in PUFA.

## 2. Materials and Methods

### 2.1. Broilers and Experimental Design

The experiment complied with Directive 2010/63/EU on the protection of animals used for scientific purposes and the experimental procedures were approved by the Ethics Committee (no. 52/30.07.2014) of National Research and Development Institute for Biology and Animal Nutrition. After the Ethics Committee’s approval, a 4-week feeding trial (14–42 days) was carried out as a randomized complete block with 4 antioxidant treatments, on 200, Cobb 500 broilers chicken, of mixed sex, established at the beginning of the experiment (♂:♀ 1:1) and obtained from local commercial hatchery. After 14 days, at the start of the feeding trial, they were weighed individually, divided into 5 groups (Control, E1, E2, E3 and E4) of 40 chicks each and housed in environmentally controlled conditions The lighting program was 16 h/day at the beginning of the experiment and a progressive increase during the experimental period until 23 h/day before slaughtering (42 days old). The experiment simulated the semi intensive system conditions, the broilers being reared on permanent wood shave litter (10–12 cm thick), in boxes of 3 m^2^ (each group was housed in a single box). They had free access to feed and water.

### 2.2. Diet Formulation

Flax meal, the oilseed raw material from the diet structure, was purchased from a local supplier and the grape pomace was purchased from the Research and Development Station for Viticulture and Vinification in Pietroasa, Buzau county (southern country). The flax meal was analyzed for the fatty acids content and grape pomace was analyzed in order to determine the resveratrol, total polyphenol concentration and antioxidant capacity. The feed’s compositions were produced at the beginning of the experiment and feed samples were collected in order to perform chemical analysis for quality control.

The diet formulations were calculated using the HYBRIMIN Futter 5 Nutrition program. The diets for all 5 groups were enriched in polyunsaturated fatty acids (PUFA) by including 4% flax meal. Compared to the control formulation Table 1, the experimental formulation included 2 varieties of dried grape pomace (GP) as natural antioxidant: 3% (E1) and 6% (E2) white grape pomace (WGP), Tămâioasă Românească variety; 3% (E3) and 6% (E4) red grape pomace (RGP), Merlot variety. The diets were manufactured on the Pilot Station of National Research and Development Institute for Biology and Animal Nutrition, Balotesti, Ilfov, Romania.

### 2.3. Broiler Slaughter

At the end of the experimental period (42 days), 6 broilers per group were randomly selected and slaughtered by cervical dislocation according to the working protocol. The last feed administration was done 24 h before slaughtering and that water was withdrawn 2–4 h before. After slaughter, the flakes, head, claws and organs were removed, in order to collect muscle tissue samples (thigh and breast meat).

### 2.4. Sampling

From the resulting chicken carcasses 6 breast and 6 thigh meat samples per group were selected. Each breast and thigh meat sample was divided in more sections to determine the color, texture parameters and oxidative stability of the meat. The samples were stored in plastic bags, labelled according to the group and broiler origin and then stored in the freezer at −20 °C until the time of determination. The muscle tissue samples thus constituted were used to evaluate the color and texture parameters of the meat. In order to carry out the lipid degradation assessment, to simplify and streamline the processing of fat extraction (obtaining homogeneous organic matrices), the samples of muscle tissue taken (6 samples of breast and thigh per group) were minced and cryogenized with liquid nitrogen at a temperature of −180 °C. Subsequent to the rapid freezing step, the samples were ground using a basic IKA A11 mill, transferred in sterile tubs and labelled, then stored in the freezer at −80 °C until determinations were performed.

### 2.5. Fatty Acids Evaluation

The fatty acid content of flax meal, grape pomace, feeds and meat samples was determined as described by Panaite et al. [24] using a gas chromatograph Perkin-Elmer Clarus 500 (Massachusetts, United States). The chromatograph has flame ionization detector (FID) and capillary separation column with a high polar stationary phase TRACE TR-Fame, (Thermo Electron, Massachusetts, United States), with dimensions of 60 m × 0.25 mm × 0.25 μm. The results were expressed in g fatty acids/100 g total fatty acid methyl esters (FAME).

### 2.6. Resveratrol Content Determination

The resveratrol content determination was performed using a liquid chromatographic method (HPLC), according to the method described by Careri et al. [25]. The chromatograph system used was a Finnegan Surveyor HPLC (Thermo-Electron Corporation—Waltham, Massachusetts, United States) and the detection was performed at 306 nm wavelength with a Photodiode Array detector (PDA). For the HPLC method was used a Hypersil Gold C18 chromatographic column (with stationary phase silica modified with octadecyl radicals), with the following characteristics: 150 × 4.6 mm and 5 μm particle size. The elution was isocratic using a mobile phase consisting of 2% acetic acid solution: methanol: acetonitrile = 60:2:38 (v/v/v). The flow rate of the mobile phase was 1 mL/min and the injection volume was 10 µL. The results were expressed in mg/100 g sample.

### 2.7. Total Polyphenols Content

The total polyphenol content of extracts was determined spectrophotometrically according to the method described by Untea et al. [26]. For the sample extraction, the Folin-Ciocalteu reagent was used and the absorption was performed at 732 nm. The results were expressed as mg gallic acid equivalents per gram of dried sample (mg GAE/g).

### 2.8. Total Antioxidant Capacity

The total antioxidant capacity of the extracts was determined using the phosphomolybdenum method [26]. The absorbance was performed at 695 nm and the results were expressed as ascorbic acid equivalents per gram of dried sample (mmol ascorbic acid equivalent/g).

### 2.9. Instrumental Color Measurements

Determination of meat color parameters in the CIELAB space by measuring the parameters L* (lightness), a* (saturation index in green/red), b* (saturation index in blue/yellow), h* (hue angle) and C* (metric chroma) and the total color difference (ΔE*) was performed according to the method described by Panaite et al. [27] using a Konica Minolta CR-400 (Tokyo, Japan) colorimeter. The results were expressed as an average for three measurements/sample.

### 2.10. pH Assessment

The pH of the samples was measured using a Hach HQ30d pH-meter (Hach, USA), according to the SR ISO 2917: 2007 standard at 24 h *post-mortem*. In order to measure the pH, a meat-water mixture was prepared from 5 g of meat which was cut into pieces with a knife and mixed with 5 mL of distilled water, at neutral pH [28], according to the method of Korkeala et al. [29] with some modifications. At least three replicate measurements for each sample were carried out and the electrode was cleaned after each measurement. Buffer solutions of pH 7 and pH 4 are used for calibration of pH meter.

### 2.11. Texture Profile Analysis

The chicken meat texture parameters determined by a double cycle compression was performed using a Perten TVT 6700 texturometer (Perten Instruments, Sweden), equipped with a Compression Platen cell. The analysis of the texture profile (TPA) resulting from the application of the compression test highlights as main texture parameters—hardness or firmness, springiness, resilience, cohesiveness and, as secondary parameter, gumminess. The thigh and breast meat samples were cut into cylindrical pieces of 10 mm thickness and 15 mm diameter, after which they were subjected to compression of 50% of the initial height. The principle of the method consisted in applying a tension with a stainless-steel cylinder, with a 20 mm diameter on the meat samples and with the automatic recording of the resistance force that they oppose to deformation. The measurements on a sample were repeated at least three times and the results were expressed as their average.

### 2.12. Evaluation of Lipid Oxidation Parameters

The oxidative stability of the meat was determined by means of primary lipid degradation parameters, dienes and conjugated trienes, respectively by secondary parameters, represented by the values of p-anisidine and those of thiobarbituric acid-reactive substances (TBARS), according to the method described by Untea et al. [26]. The lipid peroxidation parameters were spectrophotometrically determined by using a V-530 Jasco (Japan Servo Co. Ltd., Japan) spectrophotometer.

### 2.13. Principal Component Analysis

Principal Component Analysis (PCA) was made in order to evaluate the relationship between meat features. This method is useful in order to evaluate meat quality in a synthetic manner. The results were visually transposed by reducing dimensionality using two-dimensional scatter plots [23]. The measured variables at the beginning and then extracted by computer were represented by a load graph that expresses the correlation between them. A positive correlation results from close variables and a negative one if the variables are opposite to each other.

### 2.14. Statistical Analysis

All results were statistically processed for each quality parameter of the chicken samples. The analytical data were compared by analysis of variance (ANOVA and t test), using the StatView program for Windows (SAS, version 6.0). The results of this experiment are presented as mean values, the differences being considered statistically significant at *p* < 0.05 by PLSD test. The Principal Component Analysis (PCA) using the SPSS 26.0 (trial version) software for Windows (IBM, New York, USA) was applied in order to underline the relationships between the analyzed meat quality parameters and for the determination of linear correlation coefficients at *p* < 0.05 level of significance. Each bird was considered as the experimental unit. Sex was not included in the statistical model because at farm level, the broilers sexing is not practiced. This study was carried out in similar conditions at experimental level and broiler sex is not considered a decision factor.

## 3. Results

### 3.1. Chemical Composition

The flax meal, used in this study to enrich the broiler diets in fatty acids, has 64.07% PUFA of which 48.07% n-3 and 16.00% n-6, with an ideal n-6: n-3 PUFA ratio of 0.33. As natural antioxidant grape pomace of two varieties was used. The white grape pomace (WGP) was characterized by a resveratrol content of 1.99 mg/100 g dry matter (DM); an antioxidant capacity of 393.99 mmol ascorbic acid equivalent/g DM and a total polyphenol concentration of 13.13 mg GAE/g DM. The red grape pomace (RGP) had a resveratrol content of 2.25 mg/100 g DM, an antioxidant capacity of 346.36 mmol ascorbic acid equivalent/g DM and a total polyphenol concentration of 21.05 mg GAE/g DM.

### 3.2. Effect of Grape Pomace on Production Parameters of Broiler

There were no statistical differences (*p* > 0.05) regarding the production parameters Table 2 recorded during the entire experimental period (14–42 days).

### 3.3. Meat Quality Parameters of Thigh and Breast Meat

#### 3.3.1. Effect of Using Grape Pomace in Broiler Diets on Meat Color and pH

The diet supplementation with GP led to the meat color variation represented by L*, a*, b*, h*, C* and ∆E* parameters, depending on the inclusion level in diets and the grape pomace variety used Table 3.

The thigh meat lightness (L*) decreased significantly (*p* < 0.05) in E3 group compared to the control and E1 groups. Compared with the control group, the redness values increased significantly (*p* < 0.05) for E1 and E4 meat samples. A significant redness decrease was obtained in E3 group (2.17), compared with E1 group (6.74). These groups have the same GP level but from different varieties. Significant differences for redness were obtained between E3 and E4 groups with RGP but at different levels. Regarding the meat yellowness, its value increased significantly (*p* < 0.05) in E4 group, compared to the control group. The same trend was observed for chroma, which differed (*p* < 0.05) also in group E4, with the highest level of RGP inclusion, compared to the control group. The highest value for the total color difference was obtained in meat samples from group E4 with 6% RGP. The use of GP in broiler diets led to the pH value reduction of thigh meat for E1, E3 and E4 groups, compared to the control.

A significant increase (*p* < 0.05) of breast yellowness was observed for E2, E3 and E4 samples, compared to the control group. A decrease (*p* < 0.05) of chroma value in group E4, compared to the control group was found. Color difference was also visible in the case of breast meat (ΔE > 2), a higher difference being observed in E3 group, with 3% RGP.

#### 3.3.2. Effect of Using Grape Pomace in Broiler Diets on Meat Texture Parameters Determined by Double Cycle Compression

A significant increase (*p* < 0.05) of the thigh meat hardness, compared to the control group, was obtained for group E3 with 3% RGP. When including 6% RGP in diet (E4), the thigh meat hardness decreased remarkably, compared with that of the E3 group Table 4. Compared with the control group, significant differences (*p* < 0.05) regarding the thigh meat springiness was obtained when the RGP was included in diets (E3 and E4). Thigh meat from group E3 showed a higher springiness compared to that from group E4. Regarding the resilience parameter, the lowest value (*p* < 0.05) was obtained in group E3, with 3% RGP. At the same time, the thigh meat resilience was lower than that obtained in group E4.

Regarding the breast meat hardness, statistically significant differences (*p* < 0.05) were obtained between control group C and groups E1, E2 and E4. The use of 6% RGP (E4) in broiler diets led to approximately doubling of the breast meat hardness value (16.0N), compared to the hardness obtained for the control group (8.88 N). The breast meat resilience from all 4 groups (E1, E2, E3, E4) was significantly different (*p* < 0.05), compared to the values obtained in control group. The group with 3% RGP (E3) recorded the highest resilience value, which can be correlated with the low breast meat hardness. Regarding the breast meat cohesiveness, it indicated a significant increase (*p* < 0.05) in E1 and E3 groups, compared to the control group. Grape pomace level increase, regardless of variety, led to cohesiveness decrease which was significant (*p* < 0.05) between E3 and E4 groups, both having RGP in diet. Both grape pomace varieties inclusion (white and red), at different levels (3% and 6%) in broiler diets had a significant effect (*p* < 0.05) on the breast meat gumminess. The breast meat samples from 6% grape pomace groups had a lower degree of gumminess compared to those with 3% grape pomace.

#### 3.3.3. Effect of Using Grape Pomace in Broiler Diets on Lipid Oxidation

The lipid oxidation parameters of thigh meat samples are shown in Table 5. As can be seen, the dietary supplementation with flax meal and grape pomace did not induce significant (*p* > 0.05) variations of the primary oxidation parameters in thigh and breast meat. Regarding the secondary parameters, variations were evidenced for TBARS value in thigh meat. There was a significant (*p* < 0.05) decrease in all diets administered to the experimental groups, compared to the control group. The lowest value was determined in 3% WGP (E1). Similarly, significant (*p* < 0.05) differences were observed in terms of p-anisidine and TBARS values for breast meat. The p-anisidine level in the 6% WGP meat samples (E2) was found to be significantly lower (*p* < 0.05) by 44.35% compared to that found in the control group. As well, significant differences were highlighted for TBARS value in 3% white and 6% RGP supplemented groups (E1 and E4 groups) unlike to the control group.

#### 3.3.4. The Relationship between Meat Quality Parameters

**Figure 1:**
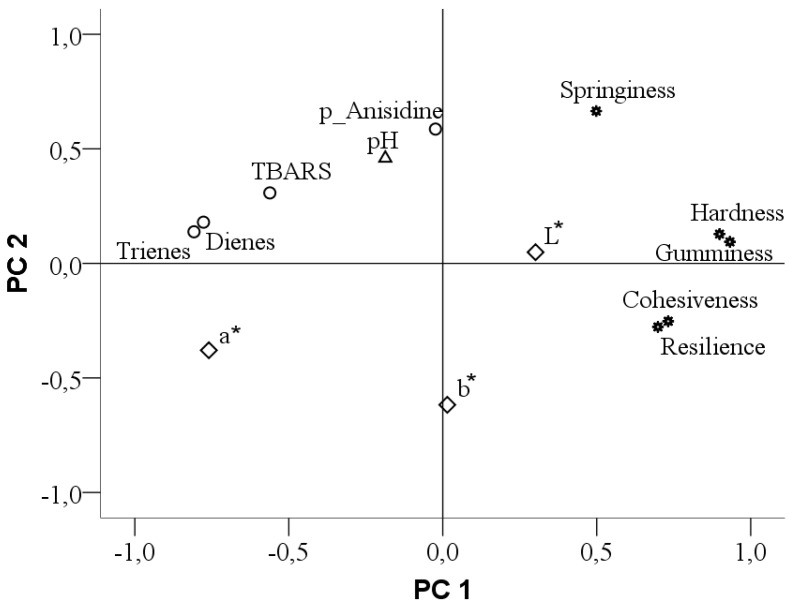
Loading plot of first two principal components based on L*, a*, b* color parameters, pH-value, textural parameters and lipid peroxidation parameters.

PCA loading of L*, a*, b* color parameters, pH-value, textural parameters and lipid peroxidation parameters is shown in Figure 1. In PCA, the first two PCs were extracted explaining 54.72% of the total variance (PC1 = 43.32%, PC2 = 11.39%) for the meat quality parameters.

The first principal component, PC1, was characterized by textural parameters (hardness, gumminess, cohesiveness and resilience), pH, color parameter (luminosity) and lipid degradation parameters (dienes, trienes and TBARS). The second component, PC2, was defined by textural parameter (springiness), pH-value, secondary lipid degradation parameter (p-anisidine) and color parameter (b*).

## 4. Discussion

The present research aimed to evaluate the effect of four diets containing different levels of grape pomace on meat quality, with attention to the color and texture parameters and the oxidative stability of meat.

### 4.1. Production Parameters

The production parameters data showed that the supplementation with grape pomace to broilers fed PUFA enriched diets did not influence the body weight, average daily feed intake or feed conversion ratio of broiler. The results of the current study are in agreement with Aditya et al. [18] and Ebrahimzadeh et al. [30]. They found that an inclusion rate of grape pomace between 5 and 10 g/kg diet did not affect (*p* > 0.05) body weight gain, feed intake or feed conversion ratio during the overall feeding-trial. Contrary to our results, Kumanda et al. [31] reported a lower feed intake and feed conversion (*p* < 0.05) when included a 7.5% grape pomace level in broiler diets but there were no dietary effects on weight gain.

### 4.2. Chemical Composition

Flaxseed meal is one of the most studied oilseeds due to its rich content of α-linolenic acid (ALA), an essential PUFA n-3 fatty acid [32] but also for linoleic acid (LA) content, n-6 essential fatty acid, these two PUFAs being essential for animals’ organisms [33]. The data recorded in this study are consistent with other published data regarding the flax meal fatty acids [24,34].

Resveratrol is a phytoalexin from the stilbene’s family, with strong antioxidant [35,36], anticancer [37] and phytoestrogenic properties [38]. It is well known that the grapevine (*Vitis vinifera* L.) has the highest concentrations of resveratrol, its distribution being in roots, seeds and grape clusters but the highest concentration is found in skin, containing about 50–100 µg/g [39]. The results obtained in the present study for the grape pomace are comparable with other published results [40] for red grape pomace samples, obtained from Romanian wines. They reported less than 5 mg/100 g of resveratrol for Fetească Neagră and Cabernet Sauvignion from Transylvania area. Similar results, ranging from 0.30 to 19.20 mg/100 g sample, were obtained in a study [41] regarding the resveratrol extraction with carbon dioxide from grape pomace. According to Fabris et al. [42], in a study regarding the antioxidant activity of trans-resveratrol (trans-3,5,4′-trihydroxystilbene) and one of its glycosylated derivatives, trans-piceid (trans-5,4′-dihydroxystilbene-3-O-β-D-glucopyranoside), these two stilbenes have the ability to inhibit the linoleic acid peroxidation and radical scavenging ability against different free radicals (DPPH) and radical initiators.

### 4.3. Meat Colour

There are a few factors that highlight the meat quality change, including its color, texture and general appearance. Meat discoloration is associated with the ability of oxidation processes and those of enzymatic reduction to maintain metmyoglobin levels in meat [43,44]. In this study, the meat color parameters determined were L*, a*, b*, h* and C*. Lightness (L*) is the amount of incident light that a surface reflects (dark or light sensation); a* represents the saturation index in green/red (redness); b* is the saturation index in blue/yellow (yellowness); h* represents the hue angle or chromatic tonality; C* is the metric chroma [45].

In the present study, the results indicated an increase of L*, a*, b* parameters. Both variety of grape pomace (E1 and E4) had a significant effect (*p* < 0.05) on thigh meat redness, compared to the control group. It was observed that the increased level of RGP led to the red meat color intensification. However, the C* values for thigh meat were lower (*p* < 0.05) in 6% RGP group (E4), compared to 3% WGP group (E1). The breast meat yellowness was higher at 6% WGP in diets. The intensity of breast meat red color (chroma, C*) decreased significantly (*p* < 0.05) in group with 6% RGP (E4), compared with the 6% WGP breast samples (E2). Generally, low fat levels and high meat water content lead to higher redness values and lower lightness values [46], as happened in the present study, for the thigh meat. At the same time, the meat lightness decrease reveals a higher sanity degree of the samples [47]. The meat yellowness increase could be related to the haem oxidation, the most common component of hemoglobin, the red pigment in blood [48]. Sayago-Ayerdi et al. [49] studied the effects of using red grape pomace (2%) in chicken burgers and the results indicated a lightness reduction attributed to the meat pigment dilution. The highest value of total color difference was obtained in meat samples from E4 group (7.16), which included 6% RGP. This fact revealed that the meat samples from E4 group were the most dissimilar in color, compared to the control samples, a result that may be due to anthocyanins from red grape pomace [50].

The fact that the breast meat is mainly composed of white muscle fibers (low in myoglobin), while the thigh meat is composed of red fibers (richer in myoglobin) justifies why the breast meat samples appear to be darker [51]. Literature studies [52,53] have shown that the redness positively influences the consumer’s perception of meat [54]. It has been reported that 1% phenols from grapes addition in meat increases the redness value but this is not perceived as negative [55]. In the present study, yellowness increased values for both breast and thigh meat samples highlighted more pronounced meat paleness. Moreover, the meat color may vary from light pink to bright red or dark red [56], so the results obtained were considered adequate. The flavonoids content from grape pomace are mainly correlated with color parameters. However, due to their strong antioxidant activity, phenols can influence meat color, acting mainly on blood myoglobin but also on lipid oxidation [50,54].

There is information in the specialty literature that confirms that using high levels of grape seed extract in diets adversely affects meat color [55]. The results obtained in this study are consistent with those reported by Brannan [57] who evaluated the effects of using 0.1% grape seed extract directly into the chicken meat (breast and thigh) on its color parameters. It was observed that the use of grape seed extract changed meat color. The lightness of the breast ranged from 77.4–79.0; redness from 2.9–4.2, significantly darker (*p* < 0.05); yellowness has been lower, from 8.1–9.8), compared to control group. Also, the data obtained in the present work are similar to those obtained in another study [58] which used an antioxidant solution (8% for 4 kg of meat). The lightness and redness of meat did not differ significantly among samples but the yellowness values were significantly higher. Kumanda et al. [59] evaluated several levels of grape pomace in Cobb 500 diets and observed that experimental diets had no effect on meat pH, L*, a*, b*, C* and h*.

### 4.4. Meat pH

Zhang et al. [60] shows that a meat with high pH (6.10–6.79) has a better stability during freezing, since it has better functional properties in terms of water holding capacity, protein solubility or yield compared to a normal pH meat (5.40–5.79). In this study, there was a slight decrease of thigh meat pH but the breast meat showed a pH increase (*p* > 0.05) proportional to the grape pomace level used in diets. Also, pH value plays an important role on meat quality parameters, such as color, texture, tenderness, juiciness and oxidative stability [61]. The pH is correlated with meat lightness, so that if the lightness value is low, respectively the meat is darker, the pH value will be high [62].

### 4.5. Texture Parameters

A higher level of RGP in diet (E4) indicates a tenderness increase in thigh meat samples. The meat hardness increase in the present study can be correlated with the gelation properties of the protein from the seeds of grape pomace, included in the diet. The water retention process through the protein matrix and the swelling in contact with water influences the texture, cohesiveness and springiness of meat, these parameters varying depending on the protein, fat and water contents of meat [45]. The results regarding meat springiness indicated variations depending on the type of grape pomace and the level of inclusion in experimental diets. It was observed that compared to the control group, a lower level of grape pomace inclusion (3%) in E1 and E3 groups caused an increase of breast meat samples cohesiveness. The same trend was observed for gumminess, although the values were significantly higher (*p* < 0.05) in all experimental groups, compared to the control. The groups with 6% grape pomace had a lower degree of gumminess compared to those with 3% grape pomace. Thus, it is possible to deduce the favorable effect of supplementing the broiler diets with grape pomace from red grapes on the breast meat gumminess parameter. The significant increase of meat hardness from E1 and E2 groups compared to the control group, suggests a tenderness decrease of breast meat. This tenderness was directly proportional to the level increase of white grape pomace in diets. The compounds present in grape pomace had a strengthening effect on the muscle fiber. It is well known that lipids influence meat, contributing to its flavor and texture, respectively to their tenderness and juiciness [63,64]. In a similar study [65] when different winery by-products (grape seed, skin and pomace) added in diet effects on chicken breast meat quality were investigated, there was no significant (*p* > 0.05) effect on the textural characteristics of meat.

The breast meat samples from 6% grape pomace groups had a lower degree of gumminess compared to those with 3% grape pomace.

### 4.6. Oxidative Stability of the Meat

Triacylglycerides, phospholipids and sterols are lipid classes from intra and extracellular space of meat, easily prone to oxidation and the main reason of rancid odor of depreciated meat [41]. Initiation, propagation and termination are the three components of lipid oxidation process and the resulted chemical compounds are primary (hydroperoxides) and secondary (aldehydes, alkanes) oxidation products [66].

The primary oxidation products are quantified as conjugated dienes and trienes and secondary oxidation products as p-anisidine and TBARS values [67]. The rate and extent of lipid oxidation are influenced by several factors, which include iron content, distribution of unsaturated fatty acids, pH and antioxidant levels [68,69]. Generally, the copper and iron ions lead to the hydroperoxides forming and separation, resulting volatile decomposition products. The conjugated compounds (dienes and trienes) measurements have the role of monitoring the oxidation process installation but cannot ensure more information regarding the structure of the compounds [66,70]. Also, the conjugated diene content is an index of lipid stability. p-Anisidine value is a secondary lipid degradation parameter, used to determine the aldehydes content from meat fat [71]. Thiobarbituric acid-reactive substances (TBARS) determination is the most used method for identifying lipid degradation. This procedure measures the malondialdehyde (MDA) content which results from carbon-carbon double bond of polyunsaturated fatty acids [72].

The obtained results indicate an improvement in thigh meat oxidative stability. The TBARS value decreased significantly (*p* < 0.05) in all experimental groups of thigh meat compared to the control. Regarding the breast meat, there was a significant (*p* < 0.05) decrease only in E1 and E4 groups, compared to the control. The TBARS assessment is the main method to quantify malondialdehyde (MDA), which is the major marker of lipid oxidation. MDA is the most important aldehydes produced during the secondary lipid oxidation of PUFA [73]. Studies have shown that the accepted limit value for which meat is not considered to be rancid is between 0.02–2.55 mg MDA/kg [74,75].

Additionally, about the secondary parameters, the new formulation diet induced a significant variation, showed that oxidation occurred slowly depending on the variety or level of grape pomace. This differentiated effect on meat samples can be justified by the antioxidant compounds of the two variety of grape pomace (white and red grapes). Previous findings proved that diets containing grape pomace delayed meat lipid oxidation. These results agree with what has been previously reported [76] in a study with grape pomace on meat lipid oxidation in chickens. The reported results indicated a linear reduction of lipid oxidation in both thigh and breast meat samples with the grape pomace level increase in diet. In a similar study [77] it was shown that using grape pomace in broiler diets did not affect (*p* > 0.05) lipid oxidation, determined as TBARS, both in thigh and breast meat. These differentiated results may be due to the chemical composition of grape pomace, respectively its antioxidant capacity. The chemical composition can be influenced by a number of factors such as grape variety, environmental and climate conditions, soil type, degree of ripening and processing procedure [78,79,80,81,82,83]. The protective effect of natural antioxidants due to their phenolic compounds is manifested by the slowing of oxidative processes. Thus, antioxidants naturally defend cells through different pathways of action such as by activating antioxidant enzymes or scavenging the free radical species [84].

### 4.7. The Relationship between Meat Quality Parameters

The result of the principal component analysis (PCA) shows a plot of the features on the two principal components Figure 1. Two groups of variables were clearly distinguished lying on the first PC far from the origin. The first group included the textural parameter, hardness, gumminess, cohesiveness and resilience. These variables are negatively correlated with the other group, the lipidic degradation parameters dienes, trienes and TBARS, lying near the first principal component, PC1 on the opposite side. The second component, PC2 essentially grouped the p-anisidine and pH as an independent cause of variation, the variable placed farthest from the origin were p-anisidine followed by pH. The p-anisidine and pH parameters are placed on opposite quadrants to the b* parameter showing that these were negatively correlated. An opposite relation can be seen between springiness and cohesiveness, which was expected. Regarding the correlations obtained, highly significant (*p* < 0.01) positive ones can be observed between textural parameters, hardness and gumminess (*r* = 0.975), cohesiveness and resilience (*r* = 0.920) which are indirectly correlated with primary lipid degradation parameters, dienes and trienes. Also, the meat lightness (L*) is negatively correlated with primary lipid degradation parameters, this fact being predictable, the lower primary lipid degradation of the samples, the higher L* parameter. A high positive correlation was found between diene and triene (*r* = 0.938). The resilience is negatively correlated with diene (*r* = −0.714) and trienes (*r* = −0.661). Significant correlations (*p* < 0.01) were also found between cohesiveness and diene (*r* = −0.666) and trienes (*r* = −0.644) and, between hardness and diene (*r* = −0.599) and trienes (*r* = −0.593). The secondary parameter of lipid peroxidation, TBARS shows a relationship with cohesiveness (*r* = −0.594), significant at *p* < 0.01. This may be explained by the fact that meat cohesiveness is influenced by the lipid peroxidation, if there is in an advanced stage, it will highly affect the meat quality, leading to cohesiveness diminishing.

## 5. Conclusions

The supplementation of broiler diets enriched in PUFA with grape pomace led to improved meat color, more evident in the thigh and texture, essential parameters for consumer’s choice. Similar results were obtained for meat hardness, significant differences being observed in all thigh samples, while the breast was significantly influenced only by red grape pomace supplementation, compared to the control. Texture profile analysis revealed that diet formulation modifications led to changes of chicken breast hardness, cohesiveness and gumminess. Also, the grape pomace supplementation indicated an improvement in the thigh meat oxidative stability, especially in regard to the TBARS value.

## Figures and Tables

**Table 1:** Ingredients and chemical composition of broiler diets.

Diet Composition %	Starter Stage (1–13 Days)	Grower Stage (14–28 Days)					Finisher Stage (29–42 Days)				
		Control	E1	E2	E3	E4	Control	E1	E2	E3	E4
Corn	32.57	35.50	31.37	37.16	31.60	37.36	39.37	34.23	31.12	34.46	31.97
Wheat	20.00	20.00	20.00	10.00	20.00	10.00	20.00	20.00	20.00	20.00	19.58
Corn gluten	2.00	4.00	6.00	9.25	6.00	8.73	6.00	6.00	10.00	6.00	10.00
Soybean meal	36.17	27.02	25.02	22.50	24.86	22.83	20.81	21.32	16.81	21.16	16.53
Flax meal	0.00	4.00	4.00	4.00	4.00	4.00	4.00	4.00	4.00	4.00	4.00
White grape pomace	0.00	0.00	3.00	6.00	0.00	0.00	0.00	3.00	6.00	0.00	0.00
Red grape pomace	0.00	0.00	0.00	0.00	3.00	6.00	0.00	0.00	0.00	3.00	6.00
Vegetable oil	3.85	4.21	5.21	5.50	5.13	5.50	4.67	6.29	6.68	6.21	6.50
Monocalcium phosphate	1.68	1.54	1.56	1.65	1.56	1.65	1.45	1.45	1.49	1.45	1.49
Calcium carbonate	1.51	1.40	1.41	1.40	1.41	1.40	1.33	1.32	1.34	1.32	1.35
Salt	0.39	0.36	0.36	0.37	0.36	0.37	0.33	0.34	0.34	0.34	0.34
Methionine	0.33	0.29	0.28	0.24	0.28	0.25	0.26	0.27	0.22	0.27	0.23
Lysine	0.30	0.41	0.47	0.55	0.48	0.54	0.48	0.47	0.60	0.48	0.61
Threonine	0.15	0.22	0.27	0.33	0.27	0.32	0.25	0.26	0.35	0.26	0.35
Choline	0.05	0.05	0.05	0.05	0.05	0.05	0.05	0.05	0.05	0.05	0.05
Vitamin-mineral premix ^1^	1.00	1.00	1.00	1.00	1.00	1.00	1.00	1.00	1.00	1.00	1.00
Calculated analysis %											
Metabolizable energy, kcal/kg	3039.79	3100.00	3100.00	3100.00	3100.00	3100.00	3200.00	3200.00	3200.00	3200.00	3200.00
Crude protein	23.00	21.50	21.50	21.50	21.50	21.50	20.00	20.00	20.00	20.00	20.00
Ether extractives	5.48	6.34	7.33	7.80	7.26	7.80	6.86	8.42	8.81	8.34	8.69
Crude fiber	3.77	3.36	3.45	3.56	3.54	3.78	3.14	3.34	3.33	3.44	3.52
Lysine	1.44	1.29	1.29	1.29	1.29	1.29	1.19	1.19	1.19	1.19	1.19
Methionine	0.69	0.63	0.63	0.62	0.63	0.62	0.59	0.60	0.58	0.60	0.59
Threonine	0.97	0.88	0.88	0.88	0.88	0.88	0.81	0.81	0.81	0.81	0.81
Tryptophan	0.25	0.20	0.19	0.16	0.19	0.17	0.17	0.17	0.14	0.17	0.14
Chemical analysis (g/100 g total FAME)											
Alpha-linolenic acid (C18: 3n3)		8.98	9.26	8.79	9.29	9.14	8.78	8.58	8.84	8.79	8.90
Linoleic acid (C18: 2n6)	-	49.46	51.00	51.28	50.97	51.06	50.50	51.53	51.85	50.55	52.68
PUFA	-	59.23	61.23	61.15	61.12	61.11	60.07	60.87	61.61	60.38	62.14
n-3	-	9.30	9.58	9.18	9.63	9.51	9.11	8.91	9.14	9.10	9.08
n-6	-	49.93	51.65	51.97	51.49	51.60	50.96	51.96	52.47	51.28	53.06

^1^ Content per kg diet: 1100000 IU vitamin A; 200000 IU vitamin D3; 2700 IU vitamin E; 300 mg vitamin K; 200 mg vitamin B1; 400 mg vitamin B2; 1485 mg pantothenic acid; 2700 mg nicotinic acid; 300 mg vitamin B6; 4 mg vitamin B7; 100 mg vitamin B9; 1.8 mg vitamin B12; 2000 mg vitamin C; 8000 mg manganese; 8000 mg iron; 500 mg copper; 6000 mg zinc; 37 mg cobalt; 152 mg iodine; 18 mg selenium. * FAME: fatty acid methyl esters.

**Table 2:** Production parameters throughout the experimental period (14–42 days).

Groups	Initial Weight (g)	Final Weight (g)	Average Daily Weight Gain (g/Broiler/Day)	Average Daily Feed Intake (g Feed/Broiler/Day)	Feed Conversion Ratio (g Feed/g Gain)
C	506.54	3136.67	94.04	149.36	1.57
E1	506.51	2966.11	91.26	148.61	1.61
E2	506.91	2999.81	91.63	146.44	1.58
E3	506.85	2937.04	90.09	145.85	1.60
E4	506.33	2995.74	89.25	140.62	1.56
SEM	3.192	38.353	1.237	4.693	0.033
*p* value	>0.9999	0.7559	0.8139	0.9826	0.9904

**Table 3:** Color parameters and the pH of the chicken meat.

Parameters	L*	a*	b*	h*	C*	ΔΕ	pH
Thigh meat							
Control	54.41 ^a^	3.42 ^b^	11.62 ^b^	0.79	12.16 ^ab^	0.00	6.25 ^a^
E1	55.44 ^a^	6.74 ^a^	12.80 ^ab^	1.33	13.16 ^a^	3.86	5.97 ^b^
E2	53.04 ^ab^	5.31 ^ab^	13.80 ^ab^	1.34	12.36 ^ab^	3.19	6.07 ^ab^
E3	50.66 ^b^	2.17 ^b^	12.18 ^b^	1.32	12.04 ^ab^	3.99	5.96 ^b^
E4	52.91 ^ab^	7.63 ^a^	17.20 ^a^	1.41	11.01 ^b^	7.16	6.04 ^b^
SEM	0.625	0.577	0.756	0.100	0.295	-	0.033
*p* value	0.0170	0.0061	0.0200	0.2786	0.0254	-	0.0284
Breast meat							
Control	53.24	1.10	13.89 ^b^	0.89	13.81 ^ab^	0.00	5.92
E1	55.35	1.75	15.90 ^ab^	0.89	12.68 ^ab^	2.98	5.90
E2	54.28	0.98	16.80 ^a^	0.81	14.34 ^a^	3.09	6.02
E3	53.11	1.56	18.21 ^a^	0.87	13.70 ^ab^	4.34	5.90
E4	55.37	1.35	17.10 ^a^	−0.50	11.84 ^b^	3.86	6.04
SEM	0.483	0.331	0.440	0.225	0.358	-	0.030
*p* value	0.4143	0.9550	0.0167	0.6456	0.1802	-	0.3758

^a,b^ Means within a column with no common superscript differ (*p* < 0.05).

**Table 4:** Texture parameters of chicken meat determined by double-cycle compression.

Parameters	Hardness (*N*)	Springiness (%)	Resilience (adm)	Cohesiveness (adm)	Gumminess (*N*)
Thigh meat					
Control	32.76 ^b^	99.63 ^b^	2.96 ^ab^	0.44	15.50
E1	35.87 ^ab^	99.73 ^ab^	3.26 ^b^	0.48	17.79
E2	39.56 ^ab^	99.64 ^ab^	3.37 ^b^	0.48	18.98
E3	44.75 ^a^	99.86 ^a^	2.80 ^a^	0.44	20.05
E4	37.65 ^ab^	99.55 ^b^	3.27 ^b^	0.45	17.40
SEM	1.765	0.035	0.072	0.008	0.957
*p* value	0.0253	0.0320	0.0352	0.4112	0.6110
Breast meat					
Control	8.88^c^	99.76	1.29^c^	0.24 ^c^	2.17 ^b^
E1	13.83 ^bc^	99.70	2.06 ^b^	0.35 ^ab^	5.01 ^a^
E2	14.10 ^bc^	99.74	1.89 ^b^	0.29 ^bc^	4.38 ^a^
E3	11.91 ^bc^	99.65	2.49 ^a^	0.40 ^a^	4.83 ^a^
E4	16.01 ^a^	99.71	1.97 ^b^	0.28 ^c^	4.36 ^a^
SEM	0.603	0.024	0.081	0.012	0.257
*p* value	0.0017	0.6530	<0.0001	<0.0001	0.0004

^a–c^ Means within a column with no common superscript differ (*p* < 0.05).

**Table 5:** The lipid degradation parameters of the broiler meat.

Parameters	Primary Parameters		Secondary Parameters	
	Conjugated Dienes (µmoli/g)	Conjugated Trienes (µmoli/g)	*p*-Anisidine Value	TBARS (mg/kg)
Thigh meat				
Control	7.52	3.49	17.95	0.23 ^a^
E1	6.15	2.96	15.55	0.15 ^b^
E2	7.23	3.22	15.27	0.18 ^bc^
E3	6.86	3.30	15.29	0.17 ^bc^
E4	6.83	3.45	18.47	0.19 ^c^
SEM	0.232	0.082	0.536	0.007
*p* Value	0.4415	0.2706	0.1281	0.0001
Breast meat				
Control	5.16	2.50	18.49^a^	0.17 ^a^
E1	4.31	2.03	14.92^ab^	0.14 ^b^
E2	4.43	2.20	10.29^b^	0.16 ^ab^
E3	4.78	2.28	15.58^ab^	0.15 ^ab^
E4	4.50	2.39	17.61^ab^	0.14 ^b^
SEM	0.274	0.099	1.038	0.019
*p* Value	0.8973	0.6621	0.0119	0.0218

^a,b^ Means within a column with no common superscript differ (*p* < 0.05).
